# *Abrus precatorius* Leaf Extract Reverses Alloxan/Nicotinamide-Induced Diabetes Mellitus in Rats through Hormonal (Insulin, GLP-1, and Glucagon) and Enzymatic (*α*-Amylase/*α*-Glucosidase) Modulation

**DOI:** 10.1155/2021/9920826

**Published:** 2021-07-23

**Authors:** Alex Boye, Victor Yao Atsu Barku, Desmond Omane Acheampong, Eric Gyamerah Ofori

**Affiliations:** ^1^Department of Medical Laboratory Science, School of Allied Health Sciences, College of Health and Allied Sciences, University of Cape Coast, Cape Coast, Ghana; ^2^Department of Chemistry, School of Physical Sciences, University of Cape Coast, Cape Coast, Ghana; ^3^Department of Biomedical Science, School of Allied Health Sciences, College of Health and Allied Sciences, University of Cape Coast, Cape Coast, Ghana; ^4^Department of Biochemistry, School of Biological Sciences, University of Cape Coast, Cape Coast, Ghana

## Abstract

**Background:**

*Abrus precatorius* is used in folk medicine across Afro-Asian regions of the world. Earlier, glucose lowering and pancreato-protective effects of *Abrus precatorius* leaf extract (APLE) was confirmed experimentally in STZ/nicotinamide-induced diabetic rats; however, the underlying mechanism of antidiabetic effect and pancreato-protection remained unknown.

**Objective:**

This study elucidated antidiabetic mechanisms and pancreato-protective effects of APLE in diabetic rats.

**Materials and Methods:**

APLE was prepared by ethanol/Soxhlet extraction method. Total phenols and flavonoids were quantified calorimetrically after initial phytochemical screening. Diabetes mellitus (DM) was established in adult Sprague-Dawley rats (weighing 120–180 g) of both sexes by daily sequential injection of nicotinamide (48 mg/kg; *ip*) and Alloxan (120 mg/kg; *ip*) over a period of 7 days. Except control rats which had fasting blood glucose (FBG) of 4.60 mmol/L, rats having stable FBG (16–21 mmol/L) 7 days post-nicotinamide/Alloxan injection were considered diabetic and were randomly reassigned to one of the following groups (model, APLE (100, 200, and 400 mg/kg, respectively; *po*) and metformin (300 mg/kg; *po*)) and treated daily for 18 days. Bodyweight and FBG were measured every 72 hours for 18 days. On day 18, rats were sacrificed under deep anesthesia; organs (kidney, liver, pancreas, and spleen) were isolated and weighed. Blood was collected for estimation of serum insulin, glucagon, and GLP-1 using a rat-specific ELISA kit. The pancreas was processed, sectioned, and H&E-stained for histological examination. Effect of APLE on enzymatic activity of alpha (*α*)-amylase and *α*-glucosidase was assessed. Antioxidant and free radical scavenging properties of APLE were assessed using standard methods.

**Results:**

APLE dose-dependently decreased the initial FBG by 68.67%, 31.07%, and 4.39% compared to model (4.34%) and metformin (43.63%). APLE (100 mg/kg) treatment restored weight loss relative to model. APLE increased serum insulin and GLP-1 but decreased serum glucagon relative to model. APLE increased both the number and median crosssectional area (×10^6^*μ*m^2^) of pancreatic islets compared to that of model. APLE produced concentration-dependent inhibition of *α*-amylase and *α*-glucosidase relative to acarbose. APLE concentration dependently scavenged DPPH and nitric oxide (NO) radicals and demonstrated increased ferric reducing antioxidant capacity (FRAC) relative to standards.

**Conclusion:**

Antidiabetic effect of APLE is mediated through modulation of insulin and GLP-1 inversely with glucagon, noncompetitive inhibition of *α*-amylase and *α*-glucosidase, free radical scavenging, and recovery of damaged/necro-apoptosized pancreatic *β*-cells.

## 1. Introduction

Diabetes mellitus (DM) is one of the metabolic syndromes characterized by chronic hyperglycemia culminating from dysregulated glucose metabolism secondary to defects in pancreatic *β*-cell function [1]. Pathologically, DM is a product of errors in glucose metabolism due to defects in pancreatic *β*-cell function which occasions either insulin insufficiency (type 1 DM) or insulin resistance (type 2 DM). Common symptoms of DM include polyphagia, polydipsia, blurred vision, chronic loss in bodyweight, gastroparesis, and polyuria [2]. Over time, unresolved hyperglycemia increases risk of microvascular complications of DM such as diabetic nephropathy, diabetic retinopathy, and diabetic neuropathy [2]. These complications occur due to extensive oxidative stress and lipid peroxidation which are the byproducts of reactions between glucose and biological molecules [3]. Extensive glycosylation of biological molecules such as DNA, proteins, and lipids leads to generation of unstable biochemical intermediates which serve as sources of reactive oxygen species (ROS) and nitrogen species (RNS). ROS and RNS are key drivers of oxidant and peroxidative reactions in various cells and tissues [3].

Epidemiologically, it is reported that DM affects over 387 million people worldwide [4]. This provisional estimate is projected to increase to 592 million by the year 2035 [5]. Initially, DM was thought to be adult-onset, but now, DM is shown to be independent of age, which clearly shows the enormity of the threat posed by DM to global health.

Conventionally, treatment of DM (types 1 and 2) involves the use of antidiabetic agents, which are grouped as noninsulin (including the biguanides (e.g., metformin), sulfonylureas (e.g., glyburide), SGLT-2 inhibitors (e.g., dapagliflozin), bile acid sequestrants (e.g., colesevelam), amylin mimetic (e.g., pramlintide), dopamine-2 agonist (e.g., bromocriptine), DPP-4 inhibitors (e.g., sitagliptin), GLP-1RAs (e.g., exenatide), thiazolidinedione (e.g., rosiglitazone), and *α*-glucosidase inhibitors (e.g., Acarbose and voglibose)) and insulin (e.g., human insulin and analogs) therapies [6]. The conventional antidiabetic therapies have been proven effective despite the fact that the use of some of them is costive and may be associated with serious side effects [7]. Cost, side effects, and the general belief that conventional drugs are synthetic and for that matter unsafe compared to herbal medicines, which are thought to be “natural” and relatively safe have contributed to the renewed interest in the use of herbs and herb-derived products for the treatment and management of human diseases including DM [8, 9]. This paradigm is quiet common in poorer tropical countries of the world, where most people rely on herbal medicine to meet their primary health care needs [8]. Also, this may probably affirm the usual assertion that use of herbs predates modern medicine and that natural products continue to serve as templates for pharmaceutical semisynthesis of new drugs [10] including antidiabetic agents. Importantly, these herbs and herb-derived therapies are used as adjunctive therapy in a manner that reflects the belief systems and ethnobotanical heritage of most local communities across Afro-Asian regions of the world. For instance many medicinal plants with ethnobotanical claims for the treatment of diabetes mellitus such as *Chrysobalanus orbicularis* [11], *Spondias mombin* and *Mangifera indica* [12], *Sesamum indicum* [13], and *Bryophyllum pinnatum* [14] have all demonstrated *α*-amylase and *α*-glucosidase inhibitory effects which is considered vital for glycemic control. It is in response to this renewed interest in the use of herbs and herb-derived products as adjunctive or alternative therapy, that the World Health Organization (WHO) together with other interest groups has recommended incorporation of indigenous healing systems, particularly herbal medicine into healthcare systems of poorer countries [15]. This recommendation has necessitated the need to verify health claims on medicinal herbs commonly used by local people to treat human diseases [16, 17]. Interestingly, *Abrus precatorius* commonly called “jequirity pea” is a medicinal herb extensively used in folk medicine of many cultures across Afro-Asian regions of the world for the treatment of human diseases [18–20]. Several reports have highlighted the traditional uses of *Abrus precatorius* leaves in the treatment and management of diabetes mellitus [21–23]. Of note, extracts from leaves of *Abrus precatorius* have demonstrated efficacy against diabetes mellitus [1] and breast cancer [24], two human diseases that have contributed significantly to global disease burden [25]. Many studies have justified the ethnopharmacological uses and other medicinal claims of *Abrus precatorius* by demonstrating experimentally its biological properties including free radical scavenging [26], reno-protection [27], neuro-protection [28], immuno-modulatory [19], anti-inflammatory [29], antilipotoxicity [30], and antiplatelet aggregation [31] just to mention but a sample.

Previously, glucose lowering and pancreato-protective effects of APLE were demonstrated experimentally in STZ/nicotinamide-induced diabetic rats to confirm its use as an antidiabetic folk medicine by local people in the western region of Ghana [1]. However, the mechanism underlying the glucose lowering and pancreato-protective effects of APLE remained poorly elucidated. Presently, mechanisms underlying the glucose lowering and pancreato-protective effects of APLE such as modulation of insulin, glucagon, and GLP-1, inhibition of enzymatic activity of *α*-amylase and *α*-glucosidase, free radical scavenging, and recovery of pancreatic *β*-cell damage and necro-apoptosis were demonstrated *in vivo* and *in vitro*.

## 2. Materials and Methods

### 2.1. Drugs and Chemicals

Alloxan monohydrate (Sigma-Aldrich Co., St. Louis, MO, USA), nicotinamide (Sigma-Aldrich Co., St. Louis, MO, USA), acarbose (LGM Pharma, USA), metformin (Bristol laboratories Ltd.), gallic acid (Merck KGaA, USA), rutin (Acros organics, USA), ascorbic acid (Carl Roth, Germany), quercetin (Alfa Aesar, UK), p-nitropheynyl glucopyranoside (pNPG) (BBI Biotech, China), Rat Insulin ELISA kit, Rat Glucagon-ELISA kit, and Rat GLP-1 ELISA kit (Wuxi Donglin Sci & Tech Development Co. Ltd., China) were used in the study. All other solvents and chemicals used in the study were of analytical grade.

### 2.2. Collection, Identification, and Authentication of *Abrus precatorius* Leaves

Fresh leaves of *A. precatorius* were collected from an uncultivated farm land at Sefwi Asawinso'A' (Bibiani-Ahwiaso-Bekwai District, Western Region, Ghana). The leaves were identified and authenticated by a qualified botanist at the herbarium unit, School of Biological Sciences, University of Cape Coast, where a voucher specimen (CC3366) was deposited as previously reported [1].

### 2.3. Preparation of *Abrus precatorius* Leaf Extract (APLE)

APLE was prepared according to a previous method [1]. Briefly, shade-dried leaves of *A. precatorius* were mechanically milled into fine powder. The powdered leaves were stored in airtight plastic containers prior to use. A 120 g quantity of the powdered leaves was soaked in 700 mL of ethanol for 48 hours and covered with cheesecloth. The mixture was stirred at least 3 times per day. On the second day, the mixture was filtered through a filter paper fitted in a Buchner funnel. The filtrate was subjected to separation using a rotary evaporator mounted on a water bath. After retrieving ethanol using the rotary evaporator, the resultant dark-green syrupy liquid was dried in a desiccator for several weeks leaving a dark-green solid extract. The extract was labelled and stored in a desiccator prior to use. APLE was continuously prepared in order to obtain enough quantities for all experiments.

### 2.4. Phytochemical Screening of APLE

APLE was screened for the presence of phytocompounds as previously reported [1].

### 2.5. Determination of Total Phenolic Content (TPC) in APLE

Total phenolic content in APLE was determined by using the Folin Ciocalteu reagent method as previously described [32] with slight modifications. A dilute concentration of APLE (250 *μ*L of 1 mg/mL) was mixed with 750 *μ*L Folin Ciocalteu reagent (1 in 10 dilutions with water) and 750 *μ*L of 7% Na_2_CO_3_. The mixtures were then made up to 5 mL with distilled water and incubated at room temperature for 2 hours for color development. Absorbance was then measured at 765 nm by using a spectrophotometer (UV-vis, T70 PG Instruments). After preparation of a standard gallic acid (0–300 *μ*g/mL) curve, TPC in APLE was extrapolated from the gallic acid calibration curve and expressed in gallic acid equivalent (GAE). All experiments were conducted in triplicates.

### 2.6. Determination of Total Flavonoid Content (TFC) in APLE

Total flavonoid content in APLE was measured per a previously described method [33] with some few modifications. Briefly, 500 *μ*L of *Abrus precatorius* plant extract (1 mg/mL) prepared in 50% ethanol was mixed with 500 *μ*L of 10% aluminium chloride (AlCl_3_.6H_2_O) and 1500 *μ*L of 10% sodium acetate (NaC_2_H_3_O_2_.3H_2_O). The reaction mixture was incubated at room temperature for 40 minutes, and absorbance was measured at 415 nm using a spectrophotometer (UV-vis, T70 PG Instruments). After preparation of a standard rutin (0-250 *μ*g/mL) calibration curve, TFC in APLE was extrapolated from the rutin calibration curve and expressed in rutin equivalent (RE). All experiments were carried out in triplicate.

### 2.7. Ethical Approval

All protocols involving animals were initially reviewed and approved by the Kwame Nkrumah University of Science and Technology (KNUST) Institutional Review Board on Animal Experimentation (FPPS/PCOL/006/2019). Also, experiments involving rats were carried out in accordance with the European Union Directives (2010/63/EU) on the use and care of animals in scientific experimentation. Animal suffering and the number of animals were reduced as much as possible in order to comply with the requirements in the 3R (Refinement, Replacement, and Reduction) recommendations [34].

### 2.8. Acquisition and Care of Experimental Animals

Healthy 7-8-week-old adult Sprague-Dawley rats of both sexes (weighing between 120 and 180 g) were purchased from the Noguchi Medical Research Institute (NMRI), University of Ghana. Rats were later transported to the Animal House of the Department of Biomedical Sciences, University of Cape Coast, Cape Coast, Ghana. Rats were housed in stainless cages (34 cm × 47 cm × 18 cm) with dry wood shavings as bedding, fed with normal rodent chow (GAFCO, TEMA), and had unrestricted access to water. Rats were maintained under ambient conditions of temperature, humidity, and normal dark/light cycle. However, these conditions were varied to suit specific requirements of some experiments. Rats were allowed to habituate under these ambient and laboratory conditions for over two weeks before use in experiments.

### 2.9. Establishment of Alloxan/Nicotinamide-Induced Diabetes Mellitus in Rats

Experimental diabetes mellitus was established in overnight-fasted normoglycemic adult Sprague-Dawley rats according to a previously described method [1, 35] with some modification. Briefly, rats were sequentially injected with nicotinamide (48 mg/kg; *ip*) dissolved in normal saline solution and maintained on ice; then, 15 minutes later, Alloxan (120 mg/kg; *ip*) reconstituted in 100 mM citrate buffer (pH 4.5) solution was administered to rats daily for 7 days. Subsequently, rats were given 5% glucose (5 mL/kg; *po*) solution daily for 7 days. To confirm establishment of hyperglycemia in rats, blood was collected from overnight-fasted rats 3-7 days post-Alloxan/nicotinamide and 5% glucose treatments using the tail-tip amputation method. Tails of rats were first wiped clean with sterile cotton dipped in 10% ethanol. Fasting blood glucose (FBG) was measured by using URIT G26 glucometer (URIT Medical Electronic Co., Ltd.).

### 2.10. Experimental Design

**Figure 1:**
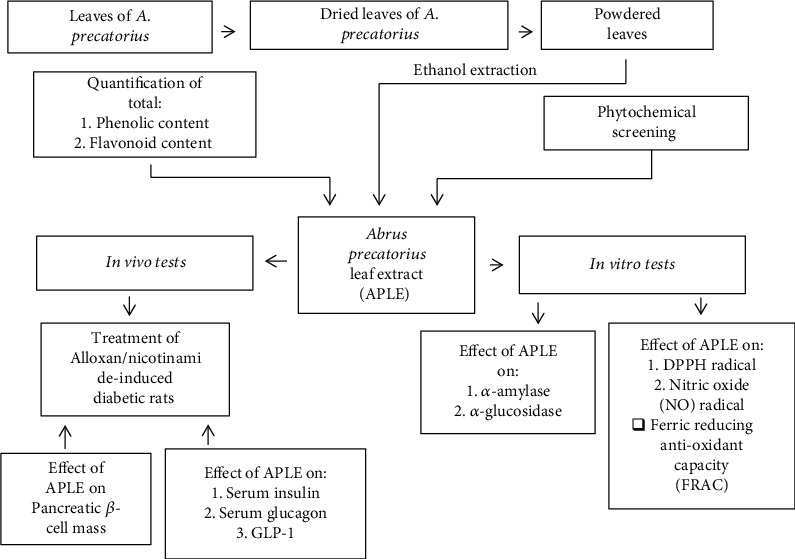
A schematic illustration of the study.

Curative rather than prophylactic treatment approach was adopted in this study, where after establishment of experimental diabetes mellitus in rats, diabetic rats were subjected to various treatments.  Figure 1 shows an outline of experiments carried out in this study. Rats having consistent FBG (11.1 mmol/L or > 250 mg/dL) upon multiple measurements were considered as having diabetes mellitus (hyperglycemia) [1, 35]. Confirmed diabetic rats were randomly assigned into five groups. Control rats were not exposed to Alloxan; instead, they were given normal saline. All groups were treated for a period of 18 days as shown below:

Control: normal saline (5 mL/rat/day; *po*) + rodent chow + water

Model: nicotinamide (48 mg/kg; *ip*) + Alloxan (120 mg/kg; *ip*) + rodent chow + water

Metformin: nicotinamide (48 mg/kg; *ip*) + Alloxan (120 mg/kg; *ip*) + metformin (300 mg/kg; *po*) + rodent chow + water

APLE (100 mg/kg): nicotinamide (48 mg/kg; *ip*) + Alloxan (120 mg/kg; *ip*) + APLE (100 mg/kg; *po*) + rodent chow + water

APLE (200 mg/kg): nicotinamide (48 mg/kg; *ip*) + Alloxan (120 mg/kg; *ip*) + APLE (200 mg/kg; *po*) + rodent chow + water

APLE (400 mg/kg): nicotinamide (48 mg/kg; *ip*) + Alloxan (120 mg/kg; *ip*) + APLE (400 mg/kg; *po*) + rodent chow + water

### 2.11. Measurement of Bodyweight

Bodyweight of rats was measured prior to the commencement of all animal experiments. Subsequently, bodyweight of rats in all groups was measured every 3 days over 18 days. Doses were adjusted to reflect changes in bodyweight every 3 days. Finally, bodyweight of rats in all groups were measured prior to their sacrifice.

### 2.12. Blood Collection, Preparation of Sera, and Isolation of Organs

After weighing rats on day 18, rats were sacrificed under deep chloroform anaesthesia. Blood samples were collected via cardiac puncture and then dispensed into labelled empty vacutainer tubes. Representative blood samples for each group were centrifuged at 3000 rpm (Eppendorf centrifuge 5702, 4°C) for 5 minutes. To obtain sera, the supernatant was collected into respective labelled sample tubes. Liver, kidney spleen, and pancreas were harvested, freshly weighed, and fixed in 10% formalin.

### 2.13. Measurement of Serum Insulin, Glucagon, and GLP-1

Serum insulin, glucagon, and GLP-1 levels were, respectively, measured by using rat insulin ELISA (Wuxi Donglin Sci & Tech Development Co. Ltd., China), rat glucagon ELISA (Wuxi Donglin Sci & Tech Development Co. Ltd., China), and rat GLP-1 ELISA (Wuxi Donglin Sci & Tech Development Co. Ltd., China) strictly according to manufacturer's instructions.

### 2.14. Determination of Median Area of Pancreatic Islets of Langerhans

After histological processing of representative pancreas, resultant photomicrographs for respective groups were scanned by using Amscope MD35 eyepiece camera as previously described [1]. Each photomicrograph was uploaded onto Adobe photoshop CS6 and subsequently superimposed on a stereological grid. The number of intersections overlying the stroma of pancreatic islets was counted. Crosssectional area of pancreatic islets of Langerhans of representative pancreas was determined by using Cavalieri method and point counting [36]. The crosssectional area was determined by the formula:

(1) $$\begin{array}{c} A=\frac{\sum P\text{ x }\left(a/p\right)}{M2}, \end{array}$$

where

*A* represents crosssectional area of pancreatic islets of Langerhans

∑*P* represents the total number of intersections overlying the stroma of pancreatic islets

*a*/*p* represents the area per point of the stereological grid

*M* represents the linear magnification.

### 2.15. Extraction of *α*-Amylase from the Pancreas of Guinea Pigs

After 20 hours of starvation, a guinea pig was sacrificed under deep anesthesia, and the pancreas was surgically removed. The pancreas was trimmed free of fat and any other tissue and immediately washed in sodium phosphate buffer (pH 7.4). After weighing the pancreas, it was kept in a freezer at 4°C. The frozen pancreas was then homogenized (1 g of pancreas: 5 mL buffer) with ice cold, 0.1 M sodium phosphate buffer (pH of 7.4) by using a homogenizer (WiseTis HG-15D homogenizer) and then centrifuged at 4400 rpm for 30 minutes at 4°C. The supernatant was pipetted into separate microfuge tubes and stored in a freezer at -20°C. To confirm the extracted enzyme (*α*-amylase), a commercially available *α*-amylase was benchmarked with the extracted enzyme (*α*-amylase) using starch and iodine. Faint or disappearance of the blue-black coloration considered as an indication of *α*-amylase activity was compared to a control set up (no enzyme + starch + iodine) where there was a deep blue-black coloration.

### 2.16. Effect of APLE on *α*-Amylase Enzymatic Activity

Inhibition of alpha (*α*)-amylase enzymatic activity was assayed per a previous method [37] with some modifications. Briefly, APLE (125 *μ*L) or acarbose at increasing concentrations (100-1500 *μ*g/mL) was added to a set of labeled test tubes containing 125 *μ*L of *α*-amylase solution in 20 mM sodium phosphate buffer (pH 6.9). The APLE or acarbose/*α*-amylase mixture was preincubated at 25°C for 10 minutes. Subsequently, 125 *μ*L of 1% starch (prepared in 20 mM phosphate buffer, pH = 6.9) was added at selected time intervals. The reaction mixture in each case was incubated at 25°C for 10 minutes. Termination of each reaction was done at time intervals by adding 250 *μ*L of 3, 5-dinitrosalicylic acid (DNSA) reagent and further heated in a water bath at 100°C for 5 minutes, then allowed to cool to room temperature. Each reaction was made up to 2.5 mL with distilled water and the absorbance measured at 540 nm using a spectrophotometer (UV-vis, T70 PG Instruments). A control set-up was prepared using the same procedure except that *α*-amylase was preincubated with distilled water instead of APLE. Alpha (*α*)-Amylase inhibitory activity was calculated as follows:

(2) $$\begin{array}{c} \%\text{inhibition}=\frac{\text{Abs control}-\text{Abs test}}{\text{Abs control}}\times 100. \end{array}$$

### 2.17. Mode of Inhibition of *α*-Amylase Enzymatic Activity by APLE

The mode of inhibition of *α*-amylase enzymatic activity by APLE was done per a previous method [38] with some modification. Briefly, 125 *μ*L of APLE (5 mg/mL) was first preincubated with 125 *μ*L of *α*-amylase solution in 20 mM phosphate at 25°C for 10 minutes in a set of labeled test tubes. Afterwards, 125 *μ*L of starch solution at varying concentrations (0.3–5 mg/mL) was added to the reaction mixtures to start the reaction. The reaction mixture was then incubated at 25°C for 10 minutes before addition of 250 *μ*L of 3, 5-dinitrosalicylic acid (DNSA) reagent to stop the reaction. The amount of product formed was assayed by measuring the absorbance at a wavelength of 540 nm using a spectrophotometer (UV-vis, T70 PG Instruments). The procedure was repeated for a control set up by preincubation of *α*-amylase with a buffer instead of APLE. Lineweaver-Burk plot from the plot of Michaeles-Menten was done using GraphPad prism version 8(Graph Pad Software, San Diego, CA, USA) for the estimation of Km and Vmax. The mode of inhibition of *α*-amylase enzymatic activity by APLE was deduced from the estimated Km and Vmax.

### 2.18. Extraction of *α*-Glucosidase from the Small Intestines of Guinea Pigs

Extraction of *α*-glucosidase from the intestines of guinea pigs was performed according to a previous method [39] with some modifications. Briefly, guinea pigs were first starved for 20 hours and then sacrificed under deep anesthesia. The small intestine located immediately above the cecum and immediately below the duodenum was surgically removed and thoroughly rinsed in ice-cold 0.02 M sodium phosphate buffer (pH 6.9) before dipping in fresh buffer solution. At a proportion of 1 g of intestine : 5 mL of buffer, the isolated intestine was homogenized (WiseTis HG-15D homogenizer, Germany) at 4400 rpm for 10 minutes at 2 minutes intermittent stoppages on ice. The homogenate was centrifuged (Eppendorf 5430R centrifuge) for 30 minutes at 4°C and the supernatant gently harvested. Aliquots were put in 2 mL cryotubes and kept at -20°C for immediate use in experiments. To confirm the extracted enzyme (*α*-glucosidase), a commercially available *α*-glucosidase was benchmarked with the extracted enzyme (*α*-glucosidase) by incubating 50 *μ*L of 3 mM p-nitrophenyl glucopyranoside (pNPG) with 25 *μ*L of commercially available *α*-glucosidase or 25 *μ*L of extracted enzyme (*α*-glucosidase) at 25°C. Observation of a yellow p-nitrophenol product after 5minutes in each case was considered a confirmation of the activity of *α*-glucosidase.

### 2.19. Effect of APLE on *α*-Glucosidase Enzymatic Activity

The inhibitory effects of APLE and acarbose (a standard *α-*glucosidase inhibitor) were assessed per a previous method [40] with some modifications. Briefly, phosphate buffer (20 mM; pH = 6.9) was used to prepare p-nitropheynyl glucopyranoside (pNPG) (substrate for *α*-glucosidase). In separate experiments, *α*-glucosidase (50 *μ*L) was preincubated with increasing concentrations of APLE (25 *μ*L in each case), acarbose, or buffer for 10 minutes in each case. Afterwards, 25 *μ*L of 3 mM p-nitrophenyl glucopyranoside was added to either APLE/*α*-glucosidase or acarbose/*α*-glucosidase preincubated mixture to start a reaction. The reaction mixture in each case was incubated at 25°C for 20 minutes. After 20 minutes of incubation, the reaction was stopped by adding 0.1 M Na_2_CO_3_ (1 mL). A control was prepared using the same procedure except that *α*-glucosidase was preincubated with a buffer instead of APLE or acarbose. The reaction product (a yellow-colored p-nitrophenol) after termination of the reaction was measured by using a spectrophotometer (UV-vis, T70 PG Instruments) at 405 nm for the determination of *α*-glucosidase activity. The results were expressed as percentage of the control. In each case, experiments were repeated three times. Percentage (%) inhibition of *α*-glucosidase activity was estimated using the formula:

(3) $$\begin{array}{c} \%\text{Inhibition}=\left(\frac{\text{Absorbance of control}–\text{Absorbance of sample}}{\text{Absorbance of control}}\right)\times 100. \end{array}$$

### 2.20. Mode of *α*-Glucosidase Inhibition by APLE

The mode of inhibition of *α*-glucosidase enzymatic activity by APLE was conducted according to a previous procedure [41] with some modifications. Briefly, in one set of test tubes, exactly 25 *μ*L of 5 mg/mL of APLE was preincubated with *α*-glucosidase solution (50 *μ*L) at 25°C for 10 minutes. In a second set of tubes, *α*-glucosidase was preincubated with 50 *μ*L of 20 mM phosphate buffer (pH = 6.9). Then, 25 *μ*L of p-nitrophenyl glucopyranoside (pNPG) at varying concentrations (0.3–3.0 mg/mL) was added to both sets of test tubes to start the reaction. The reaction mixtures were then incubated at 25°C for 20 minutes after which Na_2_CO_3_ (1000 *μ*L) was added to terminate the reaction. The amount of product released was measured spectrophotometrically using a p-nitrophenol standard curve and converted to reaction velocities, (*v*). A double reciprocal plot (1/*v* against 1/[*S*]) where [*S*] is substrate concentration was plotted. The mode of inhibition of *α*-glucosidase activity by APLE was determined by analysis of the Line weaver-Burk plot.

### 2.21. Assessment of 2, 2,-Diphenyl-1-Picrylhydrazyl (DPPH) Radical Scavenging Activity

DPPH radical scavenging activity was carried out according to a previous method [42] with some modification. Briefly, a 2 mL reaction mixture was prepared by mixing DPPH (1.0 mL of 0.135 mM) prepared in methanol and 1.0 mL of varying concentrations of APLE (40, 80, 120, 160, and 200 *μ*g/mL) or ascorbic acid. The reaction mixture was shaken vigorously and left in the dark at room temperature for 30 minutes. The absorbance was measured (UV-vis, T70 PG Instruments) at 517 nm against a blank. All tests were performed in triplicates. An equal amount of methanol and DPPH solution served as control. DPPH radical scavenging activity was estimated by using the formula:

(4) $$\begin{array}{c} \text{DPPH radical scavenging activity }\left(\%\right)=\left(\frac{\text{Absorbance of control}–\text{Absorbance of sample}}{\text{Absorbance of control}}\right)\times 100. \end{array}$$

### 2.22. Nitric Oxide (NO) Radical Scavenging Assay

NO radical scavenging activity of APLE and standards were estimated using Griess Illosvory reaction as previously described [43] with some few modifications. Briefly, Griess Illosvory reagent utilized 0.1% (*w*/*v*) napthyl ethylene diamine dihydrochloride instead of 5% 1-napthylamine. Varying concentrations (0-800 *μ*g/mL) of both APLE and standards (gallic acid and ascorbic acid) prepared and made up to 167 *μ*L were placed in labeled test tubes, and then, 667 *μ*L of 10 mM sodium nitroprusside was added. Sodium phosphate buffer (167 *μ*L, pH 7.4) was then added, and the mixture was incubated at 25°C for 150 minutes. Afterwards, 2000 *μ*L sulfanilic acid reagent (0.33% in 20% glacial acetic acid) was added and allowed to stand for 5 minutes for the completion of the reaction of diazotization. Finally, a further 2000 *μ*L of 0.1% napthyl ethylene diamine dihydrochloride was added, mixed, and was then allowed to stand for 30 minutes at 25°C. The nitrite concentration was measured (UV-vis, T70 PG Instruments) at 546 nm and was calculated with the control nitrite solution which had no APLE/or standards but having all other components of the reaction mixture. Sodium phosphate buffer was used as blank solution. Percentage (%) NO^−^ scavenging ability of APLE and standards was calculated using the formula:

(5) $$\begin{array}{c} \%\text{inhibition}=\frac{\text{Ac}-\text{At}}{\text{Ac}}\times 100, \end{array}$$

where Ac is absorbance of control and At is absorbance of test (APLE/standard).

### 2.23. Ferric Reducing Antioxidant Capacity (FRAC) Assay

FRAC was assayed per a previous method [44] with few modifications. Briefly, the reaction comprised 250 *μ*L APLE/standard solution at different concentration (12.5–200 *μ*g/mL), 625 *μ*L of sodium phosphate buffer (0.2 M at pH 6.6), and 625 *μ*L of 1% potassium ferric cyanide, [K_3_Fe(CN)_6_] into various test tubes. These were incubated for 20 minutes at 50°C to complete the reaction. Then, 625 *μ*L of 10% trichloro acetic acid (TCA) solution was added into the test tubes. The total mixture was centrifuged at 3000 rpm for 10 minutes, after which 1800 *μ*L supernatant was taken and mixed with 1800 *μ*L of distilled water. Afterwards, 360 *μ*L of 0.1% ferric chloride (FeCl_3_) solution was added and thoroughly mixed. The absorbance of the solution was measured at 700 nm using a spectrophotometer (UV-vis, T70 PG Instruments) against reaction blank. A typical blank solution containing the same solution mixture without APLE/or quercetin was incubated under similar conditions. Increased absorbance of the reaction mixture indicates increased reducing capacity. The experiment was repeated three times at each concentration. FRAC was measured as quercetin equivalent (QE).

### 2.24. Estimation of IC_50_ and EC_50_

To determine IC_50_ and EC_50_ of APLE with respective to its antioxidant, free radical scavenging, and inhibitory effect on *α*-amylase and *α*-glucosidase enzymatic activity, a series of increasing concentration of APLE was tested against the respective activities. After converting the concentrations to log_10_, it was plotted on the horizontal axis against the % maximal activities (antioxidant, free radical scavenging, and inhibitory effect on *α*-amylase and *α*-glucosidase enzymatic activity) on the vertical axis. The concentration of APLE that produced 50% of the maximal activities (antioxidant, free radical scavenging, and inhibitory effect on *α*-amylase and *α*-glucosidase enzymatic activity) assayed was determined graphically using GraphPad prism version 8 statistical software.

### 2.25. Statistical Analysis

Data obtained were presented as mean ± SD. Statistical analysis was done by using Graph Pad Prism Version 8 for Windows (Graph Pad Software, San Diego, CA, USA). Mean comparison between groups was done by using one-way analysis of variance (ANOVA) followed by Tukey's multiple comparison tests. *P* ≤ 0.05 was considered statistically significant in all analyses.

## 3. Results

### 3.1. Phytochemical Screening and Quantification of Total Phenols and Flavonoids in APLE

**Figure 2:**
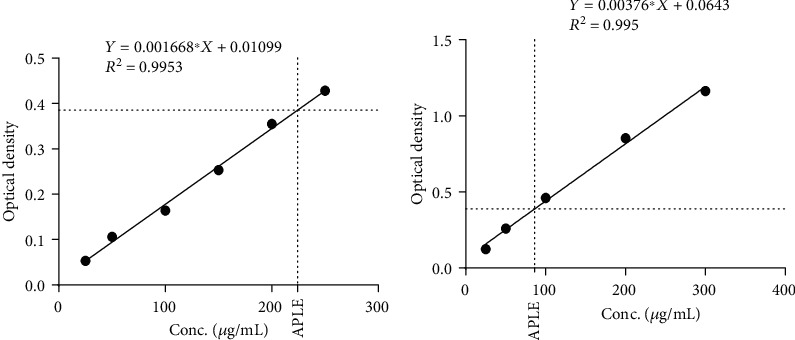
Quantification of total flavonoids and phenolics in APLE. (a) Calibration curve of rutin showing total flavonoid content in APLE. (b) Calibration curve of gallic acid showing total phenolic content in APLE. APLE: *Abrus precatorius* leaf extract.

An average yield of 9.6% APLE was obtained from the initial powdered (120 g) leaves of *Abrus precatorius*. Standard phytochemical screening showed the presence of phenols, flavonoids, tannins, alkaloids, and saponins in APLE. From the calibration curve of rutin (standard), flavonoid content in APLE was estimated to be 220.29 *μ*g/mL of rutin equivalent (RE) (Figure 2(a)). Also, from the calibration curve of gallic acid (standard), phenolic content in APLE was estimated to be 85.51 *μ*g/mL of gallic acid equivalent (GAE) (Figure 2(b)).

### 3.2. APLE Restored Loss in Bodyweight Associated with Alloxan/Nicotinamide-Induced Diabetic Rats

Compared to control rats, model rats significantly (*P* ≤ 0.05) lost bodyweight. However, treatment of Alloxan/nicotinamide-induced diabetic rats with APLE, particularly APLE (100 mg/kg), significantly restored bodyweight loss relative to model rats (Table 1). Although there were differences in the organ weight/bodyweight ratios between control and model and also between model and APLE, these differences were statistically insignificant (*P* > 0.05).

### 3.3. APLE Decreased Elevated Blood Glucose Levels of Alloxan/Nicotinamide-Induced Diabetic Rats

Sequential exposure of rats to Alloxan monohydrate (120 mg/kg; *ip*) and nicotinamide (48 mg/kg; *ip*) resulted in elevated blood glucose levels in model rats (diabetic rats) compared to control rats. Treatment of Alloxan/nicotinamide-induced diabetic rats with APLE (100, 200, and 400 mg/kg) for 18 days resulted in significant (*P* < 0.05) decrease in average blood glucose levels of diabetic rats. Over the 18-day treatment/observation period, control rats had a percentage decrease in mean blood glucose levels from the initial blood glucose level by 11.96% as against 4.3% by model rats (diabetic rats). Compared to model rats, APLE treatment, particularly APLE (100 mg/kg; *po*) produced 68.67% decrease in mean blood glucose levels from the initial blood glucose level of Alloxan/nicotinamide-induced diabetic rats over 18 days of treatment (Table 2).

### 3.4. APLE Treatment Increased the Number and Median Crosssectional Area of Pancreatic Islets of Langerhans of Alloxan/Nicotinamide-Induced Diabetic Rats

**Figure 3:**
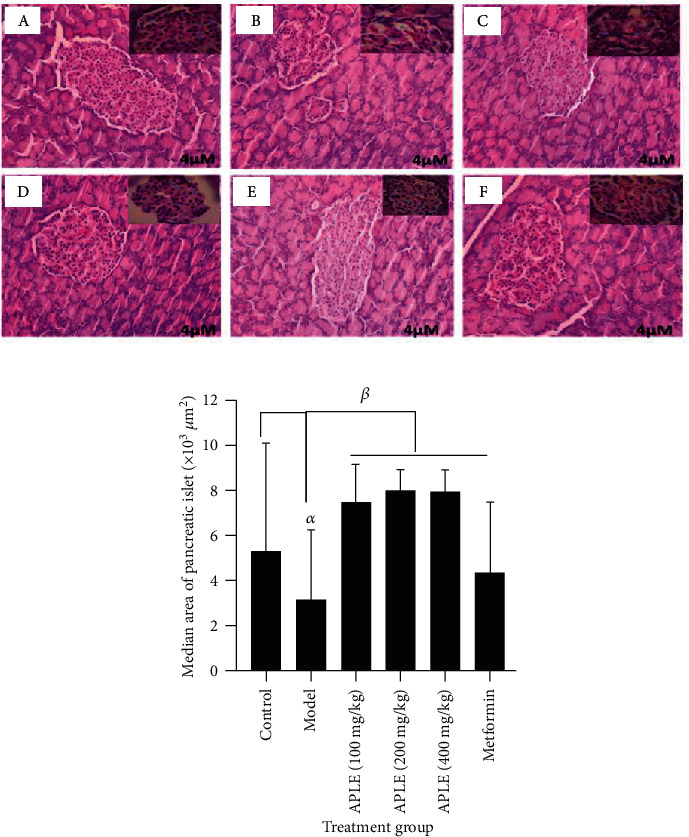
Effect of APLE and metformin on Alloxan/nicotinamide-induced pancreatic *β*-cell damage and necro-apoptosis in diabetic rats. (a) A histomicrograph of representative H&E-stained pancreatic islets of Langerhans showing (A) control, (B) model, (C) APLE (100 mg/kg *po*), (D) APLE (200 mg/kg; *po*), (E) APLE (400 mg/kg; *po*), and (F) metformin (300 mg/kg; *po*). (b) A bar graph showing the median area of pancreatic islets of Langerhans. Each bar is the mean ± SD median area of pancreatic islets of Langerhans. ^*α*^*P* ≤ 0.05 (model vs. Control); ^*β*^*P* ≤ 0.05 (APLE and metformin vs. model); APLE: *Abrus precatorius* leaf extract.

The number of pancreatic islets of Langerhans was not different between control and model rats; however, median crosssectional area of pancreatic islets of model rats was decreased compared to that of control rats. Treatment of Alloxan/nicotinamide-induced diabetic rats with APLE significantly increased both the number and crosssectional area of pancreatic islets of Langerhans compared to model rats (Figure 3 and Table 3).

### 3.5. APLE Increased Serum Insulin and GLP-1 Levels Inversely with Glucagon in Alloxan/Nicotinamide-Induced Diabetic Rats

**Figure 4:**
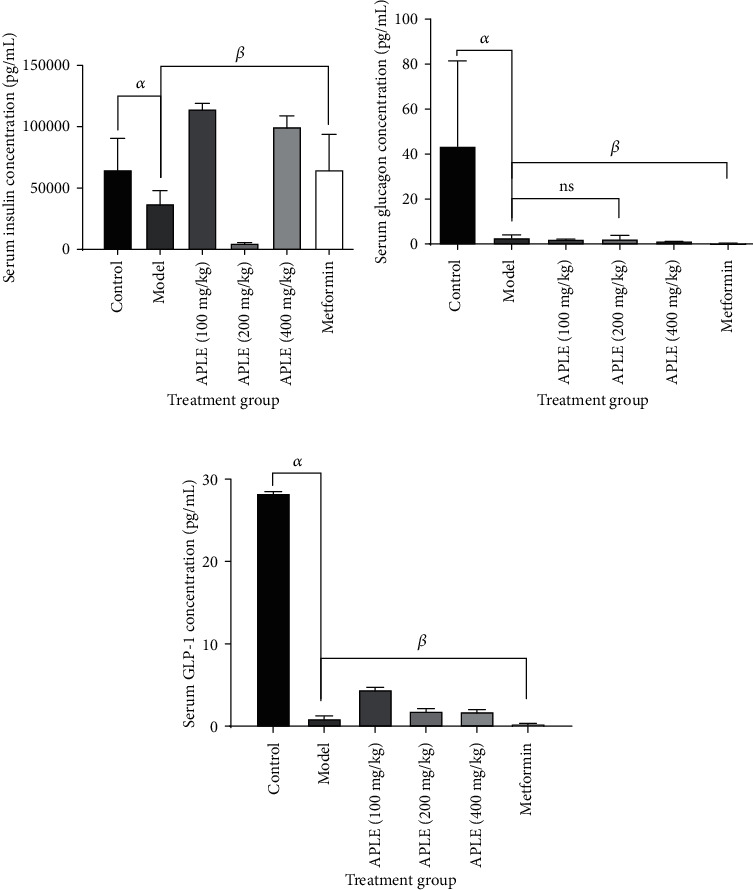
Effect of APLE on serum levels of insulin, glucagon, and GLP-1 of Alloxan/nicotinamide-induced diabetic rats. Each bar is the mean ± SD, *n* = 3. (a) Effect of APLE on serum insulin, (b) effect of APLE on serum glucagon, and (c) effect of APLE on serum GLP-1. ^*α*^*P* ≤ 0.05 (model vs. control); ^*β*^*P* ≤ 0.05 (APLE and metformin vs. model); ns: not significant; APLE: *Abrus precatorius* leaf extract; metformin (300 mg/kg; *po*).

Sequential exposure of rats to Alloxan monohydrate (120 mg/kg; *ip*) and nicotinamide (48 mg/kg; *ip*) led to decrease in serum insulin in model rats (diabetic rats) compared to control rots. However, treatment of alloxan/nicotinamide-induced diabetic rats with APLE (100, 200, and 400 mg/kg) over a period of 18 days restored serum insulin levels even more than that of control rats. Surprisingly, APLE (200 mg/kg) had no effect on Alloxan/nicotinamide-induced decrease in serum insulin (Figure 4(a)). Serum glucagon levels decreased in Alloxan/nicotinamide-induced diabetic rats compared to that of control rats. Relative to Alloxan/nicotinamide-induced diabetic rats (model rats), APLE treatment, particularly APLE (400 mg/kg), significantly (*P* < 0.05) decreased serum glucagon levels (Figure 4(b)). Serum GLP-1 significantly (*P* < 0.05) decreased in Alloxan/nicotinamide-induced diabetic rats (model rats) compared to control rats. However, treatment of Alloxan/nicotinamide-induced diabetic rats (model rats) with APLE (100, 200, and 400 mg/kg) restored serum GLP-1 levels in a dose-related manner (Figure 4(c)).

### 3.6. APLE Concentration Dependently Decreased Enzymatic Activity of *α*-Amylase

**Figure 5:**
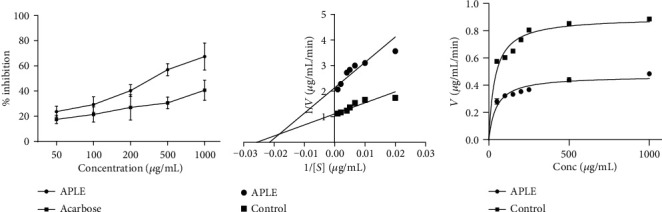
Effect of APLE on *α*-amylase enzymatic activity. Each plotted point is the mean ± SD, *n* = 3. (a) % inhibitory effect of APLE on *α*-amylase enzymatic activity, (b) Lineweaver-Burk plot showing the mode of inhibition of *α*-amylase enzymatic activity by APLE. (c) Michaeles-Menten plot showing effect of APLE on *α*-amylase kinetics (Vmax and Km). ^*α*^*P* ≤ 0.05 (APLE vs. acarbose); APLE: *Abrus precatorius* leaf extract; Vmax: maximum velocity; Km: Michaeles constant.

At equivalent concentrations, both APLE and acarbose concentration dependently inhibited enzymatic activity of *α*-amylase; however, the concentration-% inhibition curve for APLE was shifted to the left of that of acarbose (Figure 5(a)). From the Lineweaver-Burk and Michaeles-Menten plots (Figures 5(b) and 5(c)), APLE decreased maximum velocity (Vmax) of *α*-amylase/substrate reaction relative to control but increased the Michaeles constant (Km) relative to control (Table 4). Respectively, the IC_50_ estimates for APLE and acarbose were 259 *μ*g/mL and 297 *μ*g/mL (Table 5).

### 3.7. APLE Decreased Enzymatic Activity of *α*-Glucosidase

**Figure 6:**
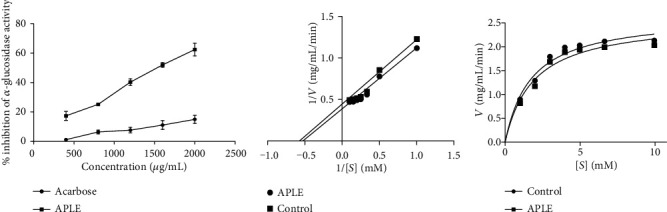
Effect of APLE on *α*-glucosidase enzymatic activity. Each plotted point is the mean ± SD, *n* = 3. (a) % inhibitory effect of APLE on *α*-glucosidase enzymatic activity, (b) Lineweaver-Burk plot showing the mode of inhibition of *α*-glucosidase enzymatic activity by APLE. (c) Michaelis-Menten plot showing effect of APLE on *α*-glucosidase kinetics (Vmax and Km). ^*α*^*P* ≤ 0.05 (APLE vs. acarbose); APLE: *Abrus precatorius* leaf extract; Vmax: maximum velocity; Km: Michaeles constant.

At equimolar concentrations, both APLE and acarbose demonstrated concentration-dependent inhibitory effects on *α*-glucosidase enzymatic activity; however, the concentration-% inhibition curve for APLE was shifted to the left of that of acarbose (Figure 6(a)). From the Lineweaver-Burk and Michaeles-Menten plots (Figures 6(b) and 6(c)), APLE decreased maximum velocity (Vmax) of *α*-glucosidase/substrate reaction relative to control but increased Michaeles constant (Km) relative to control (Table 4). Respectively, the IC_50_ estimates for APLE and acarbose were 1176 *μ*g/mL and 1090 *μ*g/mL (Table 5).

### 3.8. APLE Increased DPPH and NO Radical Scavenging Activity as well as Demonstrating Ferric Reducing Antioxidant Capacity (FRAC)

**Figure 7:**
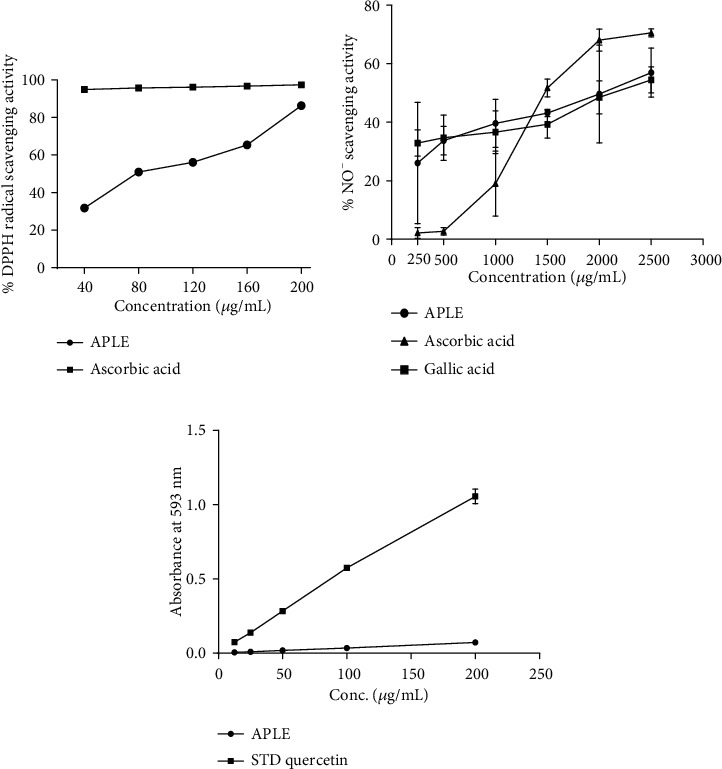
Free radical scavenging and antioxidant effects of APLE. (a) DPPH radical scavenging activity of APLE. (b) NO radical scavenging activity of APLE. (c) Ferric reducing antioxidant activity of APLE. ^*α*^*P* ≤ 0.05 (APLE vs. ascorbic acid and quercetin); APLE: *Abrus precatorius* leaf extract; DPPH: 2, 2,-diphenyl-1-picrylhydrazyl; NO: nitric oxide.

*In vitro* APLE demonstrated concentration-dependent DPPH radical scavenging activity, but it was lower than that of ascorbic acid (Figure 7(a)). Compared to ascorbic acid and gallic acid, APLE demonstrated concentration-dependent scavenging activity on nitric oxide (NO) radical. While APLE and gallic acid morphologically demonstrated a flat concentration-response (% NO radical scavenging activity) curve, ascorbic acid showed a steep concentration-response (% NO radical scavenging activity) curve (Figure 7(b)). From the IC_50_ estimates, APLE had the lowest IC_50_ value relative to ascorbic acid and gallic acid (Table 5). At equimolar concentrations, quercetin demonstrated significant concentration-dependent ferric reducing antioxidant capacity relative to APLE (Figure 7(c)).

## 4. Discussion

Herbs have been used in many capacities by mankind to improve human health and also serve as a source of natural templates for pharmaceutical semisynthesis of novel drugs. Many local communities across Afro-Asian regions of the world rely heavily on their ethnobotanical heritage to meet most of their primary healthcare needs. This study demonstrated that antidiabetic effect of APLE in experimental diabetes mellitus in rats is mediated through multiple mechanisms including inverse modulation of insulin and GLP-1 with glucagon, inhibition of *α*-amylase and *α*-glucosidase enzymatic activity, free radical scavenging, antioxidant, and recovery of necro-apoptosized pancreatic *β*-cells. As the present results corroborate earlier report on *Abrus precatorius* [1, 35], it further confirms folk claims on *Abrus precatorius* especially those made by the local communities in western Ghana, where the leaves are used to treat diabetes mellitus.

Alloxan was used as a diabetogenic agent in this study, in view of its specific pancreatic *β*-cell toxicity to establish experimental diabetes mellitus in rats. Upon exposure of Alloxan to rats, it undergoes phase 1 reaction; specifically, it is biotransformed by hepatic metabolic enzymes via reduction into dialuric acid. The dialuric acid is reoxidized back to Alloxan establishing a redox cycle leading to production of superoxide radicals (O_2_^−^), which dismutate to form hydrogen peroxide (H_2_O_2_). From the H_2_O_2_, reactive hydroxyl radicals (OH^−^) are formed through the Fenton reaction.

Resultant ROS induces increase in cytosolic calcium concentrations which in turn induces rapid destruction and necro-apoptosis of pancreatic *β*-cells. Extensive destruction of pancreatic *β*-cells occasions insulin insufficiency and hyperglycemic episode leads to glucose toxicity (gluco-toxicity). Expectedly, rats in model group (Alloxan/nicotinamide-induced diabetic rats) developed sustained hyperglycemia due to insulin insufficiency culminating from extensive destruction of pancreatic *β*-cells. However, treatment of diabetic rats with APLE over 18 days reversed the chronic hyperglycemia in diabetic rats relative to model rats (Table 2). To ascertain the mechanism by which APLE produced glucose lowering over the 18 days, serum concentrations of insulin, glucagon, and GLP-1 were measured across all groups by using a rat-specific ELISA kit. Physiologically, at any given time point, blood glucose concentration reflects a balance between glucose production (dietary sources of glucose, glycogenolysis, and gluconeogenesis) and utilization (uptake of glucose by insulin-responsive tissues and conversion of excess glucose to storage carbohydrate) primarily in response to secretion pattern and the actions of insulin and glucagon. While insulin decreases peripheral glucose concentration by increasing glucose utilization by insulin-responsive tissues such as the brain, muscles, liver, and other body cells as well as inhibition of glucagon secretion, glucagon on the other hand increases peripheral glucose levels by promoting break down of glycogen (glycogenolysis) and biosynthesis of glucose from noncarbohydrate sources (fatty acids, pyruvate, and amino acids, i.e., gluconeogenesis). Interestingly, APLE treatment reversed decreased insulin concentration inversely with glucagon in diabetic rats compared with model rats, indicating that APLE treatment improved utilization of peripheral glucose in an insulin-dependent manner. APLE-dependent increase in insulin inversely with glucagon confirms the already established observation that insulin inhibits glucagon secretion and action. To assess how APLE increased insulin but decreased glucagon in diabetic rats, the pancreatic islets of Langerhans was histologically examined; specifically, the number of islets and the median area of the islets were studied across groups. Of note, APLE treatment did not only recover partially damaged pancreatic *β*-cells but also increased the number and median crosssectional area of islets relative to that of model rats (Figure 3 and Table 3). Since insulin concentration in blood is directly related to pancreatic *β*-cell population and mass, it is possible that APLE-dependent increase in insulin inversely with glucagon was through recovery of damaged pancreatic *β*-cells as well as increase in the number of islets and mass of pancreatic *β*-cells, which enhanced insulin secretion and utilization of peripheral glucose by insulin-responsive tissues. Also, APLE produced increase in GLP-1 inversely with glucagon. GLP-1 is one of the incretins (INtestine seCRETion Insulin), and just like glucose-dependent insulinotropic polypeptide (GIP), they exert insulinotropic effect in response to the presence of glucose in the duodenum. GLP-1 and GIP are, respectively, produced by enteroendocrine L and K cells. These two hormones exert their insulinotropic effect by binding to and activating G-protein-coupled receptors (GIP receptor (GIPR) and GLP-1 receptor (GLP-1R)) in the plasma membrane of pancreatic *β*-cells. Binding and activation of the G-protein receptor leads to decoupling of the *α*-subunit of the G-protein and its transactivation of adenylate cyclase, which dephosphorylates ATP to cyclic AMP. Increase in cyclic AMP activates protein kinase A which mediates closure of K^+^ ion-gated channels. Subsequently, influx of Ca^2+^ via voltage-gated Ca^2+^ channels causes depolarization of *β*-cell membrane which eventually leads to secretion of insulin by pancreatic *β*-cells [45]. GLP-1 and GIP promote pancreatic *β*-cell proliferation, inhibit necro-apoptosis of pancreatic *β*-cells, thereby expanding pancreatic *β*-cell mass [46]. While GIP enhances postprandial glucagon response, GLP-1 suppresses postprandial glucagon response. Pancreato-protective effects of APLE could be due to enhanced release of GLP-1, since decrease in GLP-1 in diabetic rats corresponded with decreased number of islets as well as crosssectional area of pancreatic islets. Also, APLE-dependent increase in insulin could be related to GLP-1 mediation.

Enzymatically, carbohydrate digestion in humans begins in the mouth by rapid hydrolysis of both amylopectin and amylose in cooked starch by *α*-amylase, which is secreted by both salivary glands and the pancreas. Alpha (*α*)-amylase is an endoglycosidase which specifically hydrolyses internal *α*-1, 4 linkages yielding maltose, maltotriose, and *α*-dextrin. Unlike *α*-amylase, *α*-glucosidase is a brush border enzyme of duodenal enterocytes. Functionally, *α*-glucosidase hydrolyzes the terminal nonreducing (1-4) *α*-glucose residues of maltose to release a single *α*-glucose. These two enzymes serve as key targets for pharmacological modulation of carbohydrate digestion in people suffering from diseases related to errors in carbohydrate metabolism such as DM. Inhibition of these two enzymes results in significant delay in the release of glucose from disaccharides thereby reducing glucose availability and absorption. Indeed, among the available conventional oral hypoglycemic agents, *α*-glucosidase inhibitors (e.g., acarbose) enjoy therapeutic preference for the treatment of type 2 DM. Interestingly, APLE concentration-dependently inhibited these two enzymes, which reveals yet another mechanism by which APLE lowers postprandial blood glucose levels, and this observation corroborates an earlier study [47], which demonstrated that a triterpene ketone (lupenone) isolated from the leaves of *Abrus precatorius* exerted potent *α*-amylase inhibitory effect. The inhibitory effects of APLE against *α*-amylase and *α*-glucosidase mirror that of other medicinal plants known for their antidiabetic properties including *Chrysobalanus orbicularis* [11], *Spondias mombin* and *Mangifera indica* [12], *Sesamum indicum* [13], and *Bryophyllum pinnatum* [14].

Chronic hyperglycemia induces nonenzymatic glycosylation of various macromolecules leading to generation of unstable chemical species including ROS. ROS inhibits glyceraldehyde-3-phosphate dehydrogenase (GADPH) in the glycolytic pathway, thereby increasing upstream intermediates of GADPH. These glycolytic intermediates (glucose, fructose-6-phosphate, and glyceraldehyde-3-phosphate) are shunted into other biochemical pathways which are implicated in DM. Also, ROS is implicated in lipid peroxidation and oxidative stress. Oxidative stress induced by ROS as a result of chronic hyperglycemia plays a key role in the onset of various diabetic complications including insulin resistance and pancreatic *β*-cell dysfunction. In this study, free radical scavenging activity of APLE was assessed by using DPPH and NO assays, while antioxidant capacity of APLE was assessed using FRAC. DPPH is a stable free radical as a result of the delocalization of electrons all over the molecule. Delocalization of electrons in DPPH results in a deep violet color, and upon reduction by any hydrogen or electron donor, the violet color of DPPH fades and leads to formation of a pale-yellow hydrazine. The color change reflects shifting of wavelength in the visible spectra from 517 nm to 330 nm. As a result, free radical scavenging activity corresponds to reduction of DPPH, which can be quantified by measuring absorbance at 517 nm [48]. Similarly, NO partakes in a series of reactions leading to decrease in mitochondrial ATP and aconitase, which in turn induce increase in xanthine oxidase. Nitric oxide (NO) donors such as STZ promote reaction between superoxide (O_2_^−^) and hydrogen peroxide (H_2_O_2_), which yields reactive hydroxyl (OH^−^) and nitro radicals that cause DNA damage of pancreatic *β*-cells. Conversion of ferric (Fe^3+^) to ferrous (Fe^2+^) by donation of an electron by an electron donor (antioxidant agent) forms the basis of FRAC [49]. Therefore, FRAC assay provides a direct measure of the reducing or electron donating ability of an antioxidant. In this study, APLE demonstrated concentration-dependent scavenging activity against DPPH and NO and also demonstrated reducing capacity in the FRAC assay. These observations point to the ability of APLE to mop-up unstable chemical intermediates generated by Alloxan exposure to rats, thereby preventing ROS-mediated cell damage and necro-apoptosis, which accounted for extensive damage of pancreatic *β*-cells and attendant hyperglycemia in model rats. The antioxidant and free radical scavenging effects of APLE are attributable to the bioactive secondary plant metabolites identified in APLE, particularly the phenolic compounds (Figure 2). Phenolic compounds derived from plants exhibit many biological properties which account for their health benefits and a justification for their use in food and drug discovery industries. Phenolic compounds exert their biological effects by interacting with diverse cellular components including membrane transporters, protein kinases, catechol-O-methyltransferases, membrane-bound NADPH oxidases, xanthine oxidase, cyclo-oxygenases, lipoxygenases, and some transition metals [50–52]. Phenolic compounds exert antioxidant and free radical scavenging activities either directly or indirectly. Mostly, antioxidant effects of phenolic compounds are exerted indirectly by the ability of phenolic compounds to induce cellular events that lead to production of ROS-scavenging enzyme systems *in vivo*, while direct antioxidant effects of phenolic compounds are related to their ability to suppress the initiation step needed for generation of oxidant species or direct interaction with these unstable chemical species. Phenolic compounds derived from many plants have demonstrated inhibitory effects on activity of *α*-amylase and *α*-glucosidase [53–55], which supports the assertion that the inhibitory effects of APLE against activity of *α*-amylase and *α*-glucosidase observed in the present study could be due to the phenolic compounds detected in APLE. Also, tannins, saponins, and alkaloids were identified in APLE confirming a previous report [1]. Further, it is suspected that inhibitory effects of APLE on *α*-amylase and *α*-glucosidase enzymatic activity could be due to the combined effects of its phytoconstituents including tannins and saponins whose inhibitory effects against *α*-amylase and *α*-glucosidase have already been established [56–58].

Putting together this study has demonstrated that glucose lowering and pancreato-protective effects of APLE is mediated through multiple mechanisms including hormonal modulation, enzyme inhibition, free radical scavenging, antioxidant activity, and repair of damaged pancreatic *β*-cells. This study could have benefited from investigating the effect of APLE on the counterregulatory hormonal systems particularly, the catechol amines, and the stress hormone (cortisol) in gluconeogenesis (a major contributor to peripheral glucose) as well as effect of APLE on specific glucose transporters; nonetheless, the present results provide compelling basis for further mechanistic elucidation of antidiabetic effects of APLE.

## 5. Conclusion

Increase in insulin and GLP-1 inversely with glucagon, inhibition of *α*-amylase/*α*-glucosidase enzymatic activity, free radical scavenging, antioxidant, and pancreatic *β*-cell recovery underpin antidiabetic effects of *Abrus precatorius* leaf extract (APLE), and these pharmacological effects are attributable to phenolic and flavonoid contents of APLE. As this finding confirms folk use of APLE as an antidiabetic herbal medicine by local communities, it also lays a foundation for possible translational studies on APLE.

## Figures and Tables

**Table 1:** Effect of APLE on organ weight/bodyweight ratios and bodyweight change.

	Organ weight/body weight ratio				Body weight (g)		
	Spleen	Liver	Kidney	Pancreas	Initial	Final	% change in body weight
Control	0.005 ± 3.7 × 10^−5^	0.043 ± 3.0 × 10^−4^	0.005 ± 3.2 × 10^−4^	0.004 ± 2.6 × 10^−5^	153.6 ± 14.5	154.5 ± 17.4	0.58
Model	0.005 ± 1.6 × 10^−4^	0.032 ± 1.0 × 10^−3^	0.004 ± 1.3 × 10^−4^	0.005 ± 1.6 × 10^−4^	167.4 ± 23.8	156.4 ± 8.0	-6.57^a^
Metformin (mg/kg)	0.005 ± 1.2 × 10^−4^	0.042 ± 1.0 × 10^−3^	0.004 ± 1.0 × 10^−4^	0.002 ± 5.5 × 10^−4^	167.2 ± 52.9	163.1 ± 11.2	-0.02^ns^
*APLE (mg/kg)*							
100	0.006 ± 1.6 × 10^−4^	0.050 ± 1.0 × 10^−3^	0.006 ± 1.6 × 10^−4^	0.005 ± 1.4 × 10^−4^	138.5 ± 32.4	145.5 ± 62.6	5.10^b^
200	0.006 ± 2.3 × 10^−4^	0.055 ± 2.0 × 10^−3^	0.006 ± 2.4 × 10^−4^	0.004 ± 1.4 × 10^−4^	146.6 ± 43.5	140.9 ± 21.5	-3.86^ns^
400	0.005 ± 2.3 × 10^−4^	0.031 ± 1.0 × 10^−3^	0.003 ± 1.3 × 10^−4^	0.005 ± 2.0 × 10^−4^	156.2 ± 23.0	154.6 ± 24.9	-0.99^ns^

^∗^Received normal saline; APLE: *Abrus precatorius* leaf extract (APLE); ns: not significant; ^a^*P* ≤ 0.05 (model vs. control); ^b^*P* ≤ 0.05 (APLE and metformin vs. model).

**Table 2:** Effect of APLE on elevated blood glucose levels of Alloxan/nicotinamide-induced diabetic rats.

Treatment groups	Day of blood glucose peak	Blood glucose level after Alloxan/nicotinamide exposure (mmol/L)	Blood glucose level on day 18 (mmol/L)	Mean decrease in blood glucose	% decrease in blood glucose
^∗^Control	9	4.60 ± 0.282	4.05 ± 0.212	0.55 ± 0.07	11.96
Model	12	21.90 ± 9.050	20.95 ± 5.586	0.95 ± 3.464	4.34^a^
Metformin (300 mg/kg)	12	18.45 ± 1.626	10.40 ± 5.515	8.05 ± 3.889	43.63^b^
*APLE (mg/kg)*					
100	9	16.60 ± 4.52	5.20 ± 1.414	11.4 ± 3.106	68.67^b^
200	12	16.90 ± 2.969	11.65 ± 1.242	5.25 ± 8.273	31.07^b^
400	15	17.10 ± 2.828	16.35 ± 0.070	0.75 ± 2.758	4.39^ns^

^∗^Received normal saline; ^a^*P* ≤ 0.05 (model vs. control); ^b^*P* ≤ 0.05 (APLE and metformin vs. model); APLE: *Abrusprecatorius* leaf extract; ns: not significant.

**Table 3:** Effect of APLE on number of pancreatic islets and median pancreatic area of Alloxan/nicotinamide-induced diabetic rats.

Treatment groups	Number of islets	Median area (×10^3^*μ*m^2^; Q1-Q3)	Average rank
Control	16	5.299 (2.047-9.996)	65.0
Model	16^ns^	3.131 (1.445-6.142)^a^	34.9
Metformin (300 mg/kg)	18^ns^	4.336 (2.348-7.587)^ns^	50.6
*APLE (mg/kg)*			
100	27^b^	7.467 (5.299-9.635)^b^	76.0
200	25^b^	7.949 (6.624-9.153)^b^	83.2
400	35^b^	7.949 (6.744-9.394)^b^	80.3

^a^ *P* ≤ 0.05 (model vs control); ^b^*P* ≤ 0.05 (APLE and metformin vs. model); APLE: *Abrus precatorius* leaf extract; ns: not significant.

**Table 4:** Effect of APLE on kinetics of *α*-amylase and *α*-glucosidase.

	*α*-Amylase		*α*-Glucosidase	
	Vmax	Km	Vmax	Km
Control	0.8992	38.86	2.665	1.750
APLE	0.4685	46.52	2.577	1.901

APLE: *Abrus precatorius* leaf extract.

**Table 5:** Estimated IC_50_ values for APLE and standards.

	Drugs and standards	IC_50_ (*μ*g/mL)
FRAC	APLE	2.799^∗^
	Quercetin	3.243^∗^

DPPH radical scavenging activity	APLE	115.100
	Ascorbic acid	107.900

NO radical scavenging activity	APLE	1100.000
	Gallic acid	1670.000
	Ascorbic acid	1236.000

*α*-Glucosidase enzymatic activity	APLE	1090.000
	Acarbose	1176.000

*α*-Amylase enzymatic activity	APLE	297.000
	Acarbose	259.100

^∗^Values represent EC_50_; APLE: *Abrus precatorius* leaf extract; DPPH: 2, 2,-diphenyl-1-picrylhydrazyl; FRAC: ferric reducing antioxidant capacity; NO: nitric oxide.

## Data Availability

All data used to support the findings of this study are available from the corresponding author upon request.

## Abbreviations

APLE: *Abrus precatorius* leaf extract
AscAE: Ascorbic acid equivalent
DM: Diabetes mellitus
DPPH: 2, 2-diphenyl-1-picrylhydrazyl
GADPH: Glyceraldehyde-3-phosphate dehydrogenase
QE: Quercetin equivalent
IR: Insulin resistance
ROS: Reactive oxygen species.
